# Predictors of Rebleeding in Non-variceal Upper Gastrointestinal Bleeding of Peptic Ulcer Etiology in Kashmiri Population

**DOI:** 10.7759/cureus.33953

**Published:** 2023-01-19

**Authors:** Shaheena Parveen, Altaf H Shah, Showkat A Zargar, G M Gulzar, Jaswinder S Sodhi, Mushtaq A Khan, Nisar Ahmad Syed, Nazir A Dar

**Affiliations:** 1 Department of Gastroenterology, Sher-i-Kashmir Institute of Medical Sciences (SKIMS), Srinagar, IND; 2 Department of Medical Oncology, Sher-i-Kashmir Institute of Medical Sciences (SKIMS), Srinagar, IND; 3 Department of Radiation Oncology, Sher-i-Kashmir Institute of Medical Sciences (SKIMS), Srinagar, IND

**Keywords:** non-variceal, endoscope, ulcer, rebleed, gi bleed

## Abstract

Background

Upper gastrointestinal bleeding (UGIB) represents a substantial clinical and economic burden and rebleeding is one of the most important predictors of morbidity and mortality. Identifying patients who are likely to rebleed is a critical component of effectively managing patients with bleeding peptic ulcers. So, the study was undertaken to look for predictors of rebleeding in patients with bleeding peptic ulcers and try to find out the new scoring system to predict rebleeding in our population.

Material and methods

A retrospective analysis of prospectively maintained hospital data of UGIB patients was done and 480 patients of endoscopically documented peptic ulcers whose complete data was available were taken for study.

Results

Among the studied patients, men constituted 84.6%, and most of the patients were in the third to sixth decade of life with a mean age of 40.9±15.9 years, 76% were from rural areas. Only males with a mean age of 38.4±19.8 rebled with a rebleeding rate of 2.9% only. Half of the patients who rebled were in shock at the time of presentation. Those who rebled received more units of blood transfusion (mean 3±1.8), had a large mean ulcer size of Forest class IIa and IIb and epinephrine injection monotherapy group with varied statistical significance. Among rebleeders (n=14), eight patients were managed by a second endoscopic therapy, and six (42.8%) rebleeders and 1.25% of patients in total needed surgery. Two patients ultimately died giving overall mortality of 0.4% and mortality of 14.3% among rebleeders.

Conclusion

Our study found a very low rebleeding rate and mortality which could be explained by a young population with fewer co-morbidities and better response to proton pump inhibitor therapy. The significant parameters related to rebleeding were shock at presentation, degree of smoking, units of blood transfused, ulcer size, and high-risk endoscopic stigmata.

## Introduction

Upper gastrointestinal bleeding (UGIB) represents a substantial clinical and economic burden [1]. Bleeding is self-limited in 80% of patients with UGIB, even without specific therapy. Of the remaining 20% who continued to bleed or rebleed, the mortality rate is 30% to 40%. Patients at high risk for continuous bleeding or rebleeding potentially can benefit the most from acute medical, endoscopic, and surgical therapy [2]. Rebleeding is one of the most important predictors of death from UGIB and influences other important endpoints such as transfusion requirement, need for surgery, and length of hospital stay. Identifying patients who are likely to rebleed is a critical component of effectively managing patients with bleeding peptic ulcers. Triaging these patients to higher levels of care and managing them more aggressively may improve clinical outcomes [3].

Various scoring tools have been devised to identify patients with non-variceal bleeding at the greatest risk for mortality and rebleeding. These tools could be used to triage patients to a higher level of hospital care or more urgent endoscopy. They can be divided into those that use purely clinical parameters available on patient’s presentation to the hospital and those that incorporate clinical parameters and endoscopic findings. The Blatchford [4] and pre-endoscopic Rockall scores use only clinical and laboratory data to identify patients who require intervention whereas the complete Rockall score [5] also uses endoscopic variables to predict rebleeding or mortality.

Furthermore, studies have shown that parietal cell mass is much lower in Indian patients than those in western countries [6]. Also, there is an increased response of Indian patients to proton pump inhibitors (PPIs) compared to those in the west studied by Lanas et al. [7]. We undertook this study to look for predictors of rebleeding and to propose a new scoring system in non-variceal UGIB of peptic ulcer etiology in the Kashmiri population.

## Materials and methods

A retrospective analysis of prospectively maintained hospital data of UGIB bleeders was done and 480 patients of endoscopically proven peptic ulcers whose complete data was available were taken for analysis. The study was approved by the institutional ethics committee (IEC number 246).

The patient population management protocol

All patients admitted to this hospital with UGIB manifested in the form of hematemesis and/or malena or both are managed as per the departmental protocol. After receiving the patient in the emergency department and checking vital parameters; the patient is resuscitated, and complete history and clinical examination are recorded. Those patients suspected to have UGIB because of peptic ulcer are given pantoprazole 80 mg intravenous stat followed by 8mg per hour continuous infusion which is subsequently stopped or continued as per endoscopic findings. After discussing the endoscopic therapy with the patients and/or their relatives, written informed consent is obtained. All patients undergo endoscopy within 12 hours (max. of 24 hrs) of presentation and preferably immediately after resuscitation in patients with massive bleeding or shock. Endoscopic hemostasis is achieved using epinephrine injection or epinephrine injection in combination with heat probe coagulation or hemoclip application at the discretion of the endoscopist. Epinephrine (1:10,000 diluted in normal saline) is injected in aliquots of 0.5-1.0 mL into and around the bleeding area. Heat probe thermocoagulation is given to ulcers using the Olympus heat probe unit. The energy output of the heat probe is set at 25 Joule and co-aptive pulses (minimum of three) are applied until cavitation and adequate coagulation are obtained. In the case of an adherent clot, removal of the clot is tried either by vigorous flushing or snaring at the discretion of the endoscopist. Endoscopic therapy is given either after clot removal or with an intact clot. At the time of endoscopy, the size of the ulcer is estimated by placing biopsy forceps alongside the ulcer, the fully opened cup of forceps is approximately 5mm in diameter. Patients with low-risk stigmata ulcers in the form of clean-based ulcers or flat pigmented spots are discharged from the hospital and are asked to report back in case of fresh hematemesis, malena, or cold sweats associated with postural symptoms. Those with high-risk stigmata are observed in a high-care facility of the gastroenterology ward and they continue pantoprazole 8mg per hour infusion for 72 hours. Patients’ vital signs are checked every hour during the first 12 hours, every two hours for the next 12 hours, and four hours thereafter until they were discharged. The hemoglobin level and hematocrit are checked at least once daily, and a blood transfusion is given if the hemoglobin levels drop to 7g/dL or less or any time when vital signs deteriorate.

During the whole period, patients are closely observed for rebleeding which is defined by fresh hematemesis, malena, or both with either shock (systolic blood pressure of 100 mmHg or less, or a pulse rate of 100 per minute or more accompanied by cold sweats, pallor, or oliguria); or a fall in hemoglobin of 2 g/dL or more over 24 hours after initial stabilization of vital signs. Patients meeting these criteria undergo emergency endoscopy within four hours to confirm the rebleeding diagnosis. Rebleeding was managed with repeat endoscopic therapy as before.

Those confirmed to have bleeding from the stomach or duodenal ulcer by having active bleeding (spurting hemorrhage, oozing hemorrhage) or stigmata of recent hemorrhage (non-bleeding visible vessel or adherent clot) or ulcer with flat pigmented spot and clean-based ulcer without another obvious source of bleed constituted the study group. Patients with severe coagulopathy (prothrombin time 30% more than normal) and platelet count < 50,000/cmm and patients bleeding from a suspected malignant ulcer, and terminally sick patients were excluded from this study.

Indication for surgery

1. Failure of endoscopic re-treatment, i.e., those patients who continued to bleed despite second endoscopic therapy.

2. Those who rebled after the second endoscopic therapy.

Follow-up protocol

After three days, all patients received pantoprazole 40mg orally once daily for six weeks; those positive for Helicobacter pylori (H. pylori) are treated with triple therapy. Patients undergo repeat endoscopy if clinically indicated for duodenal ulcer. For stomach ulcers, repeat endoscopy is done approximately after eight weeks to look for healing of the ulcer and or biopsy if clinically indicated.

Statistical methods

Data were analyzed using the statistical package for social sciences (SPSS) version 19 (SPSS Inc., Chicago, IL) using standard analytical and statistical methodology accepted for observational studies. Number and percentage were calculated for categorical data. Pearson’s Chi-square test and Fisher’s exact test were applied to examine the difference between proportions. For continuous quantitative data, values were expressed as mean ± standard deviation. Data were checked for normal distribution with the Kolmogorov-Smirnov test. For normally distributed data, an unpaired t-test was applied; for skewed data, the Mann-Whitney U test was used to compare the difference between means. Two-tailed tests were performed and a P-value of less than 0.05 was considered significant. Multivariate analysis was done using logistic regression analysis.

## Results

This study was conducted on 480 consecutive patients. Men constituted 84.6% of studied patients. Most of the patient population were in the third to sixth decade of life; 114 (23.7%), 95 (19.8%), 84 (17.5%), and 69 (14.3%), respectively, were in the third, fifth, sixth, and fourth decade of life in decreasing order of frequency. Most of the studied subjects were from rural areas. Out of the total 480 patients, 361 (75%) were from rural areas and only 119 (25%) were from urban areas as shown in Table 1.

**Table 1:** Socio-demographic characteristics of the study population.

Characteristic		Numberof Subjects	Male n (%)	Female n (%)	Chi-square	P-value
Age group (years)	< 20	40	33(8.1%)	7(9.5%)	7.4	0.284
	20-29	114	89(21.9%)	25(33.8%)		
	30-39	69	58(14.3%)	11(14.9%)		
	40-49	95	82(20.2%)	13(17.6%)		
	50-59	84	75(18.5%)	9(12.2%)		
	60-69	47	40(9.9%)	7(9.5%)		
	70 & above	31	29(7.1%)	2(2.7%)		
Residence	Rural	361	305(84.5%)	56(15.5%)	0.010	0.919
	Urban	119	101(84.9%)	18 (15.1%)		

Only 14 out of 480 patients rebled giving a rebleeding rate of 2.9%. The mean age was 38.4±19.8 years and 40.9±15.8 years in rebleeding and non-rebleeding groups respectively which was not statistically significant. All the rebleeders were males. Overall, 19.8% of patients were smokers and only eight (1.7%), 50 (10.4%), 14 (2.9%), and one (0.2%) of patients were taking alcohol, non-steroidal anti-inflammatory drugs (NSAIDs), antiplatelet and anticoagulants, respectively, with none significantly associated with rebleeding. The studied population had a fewer number of co-morbidities with mean of 2±0 and 1.6±0.8 co-morbidities in the rebleed and non-rebleed groups, respectively. Co-morbidities seen in our patients were hypertension (8.3%), diabetes mellitus (DM) (3.3%), chronic kidney disease (CKD) (1%), stroke (6%); malignancy (0.62%), and chronic obstructive pulmonary disease (0.8%) as shown in Table 2. 

**Table 2:** Comparison of demographic and co-morbidities between rebleed and no rebleed groups. NSAID, non-steroidal anti-inflammatory drugs; CAD, coronary artery disease; DM, diabetes mellitus; CKD, chronic kidney disease; COPD, chronic obstructive pulmonary disease; SD, standard deviation.

Characteristic	All patients (n=480)	Rebleed group (n=14)	Non Rebleed group (n=466)	P-value
Age (Mean ±SD)	40.9 ± 15.9	38.4±19.8	40.9 ± 15.8	0.56
Male Sex, n (%)	406 (85%)	14 (100%)	392 (84%)	0.1
Urban domicile, n (%)	119 (25%)	4 (29%)	115 (25%)	0.74
Smokers	95(19.8%)	3(21.4%)	92(19.7%)	0.8
Mean No. of Cigarettes Per Day ± SD	8.6 ± 3.6	16.7 ± 11.5	8.3 ± 2.9	0.000 (R= 0.41)
Alcohol intake	8(1.7%)	1(7.1%)	7(1.5 %)	0.2
NSAID	50(10.4%)	0(0%)	50 (10.7 %)	0.21
Antiplatelets	14(2.9 %)	0(0%)	14(3%)	0.65
Anticoagulants	1(0.2%)	0(0%)	1 (0.2%)	0.97
All Co morbidities	52(10.8%)	2(14.3%)	50(10.7%)	0.67
Mean No. of Comorbidities SD	1.7 ± 0.8	2±0	1.6±0.8	0.5
Hypertension	40 (8.3%)	1(7%)	39 (8.4 %)	0.67
CAD	12(2.5 %)	0(0%)	12(2.6%)	0.69
Malignancy	3(0.62%)	0(0%)	3 (0.6%)	0.91
DM	16(3.3%)	2(14%)	14(3%)	0.12*
CKD	5(1.0%)	1(7%)	4(0.8%)	0.34**
Stroke	3(0.6%)	0(%)	3(0.6%)	0.91
COPD	4(0.8%)	0(0%)	4(0.8%)	0.88

The main clinical presentation of our patients was malena (71%), hematemesis + malena (17%), and hematemesis (11.3%). Seven out of 14 (50%) patients who rebled were in shock at the time of presentation in comparison to only 76 out of 466 patients (16.3%) in the non-rebleed group. Mean hemoglobin (Hb.), hematocrit, and creatinine at presentation were 9.2±2.4 g/dL, 28.5±7.1, and 1.6±0.33 mg/dL, respectively, which were not significantly associated with rebleeding as shown in Table 3.

**Table 3:** Comparison of clinical parameters between rebleed and no rebleed groups.

Characteristic	All patients (n=480)	Rebleed group (n=14)	Non Rebleed group (n=466)	P-value
Hematemesis	54(11.3%)	2(14.3%)	52(11.2%)	0.08
Malena	341(71%)	7(50%)	334(71.7%)	0.72
Hematemesis +Malena	85 (17.7%)	5(35.7%)	80(17.1%)	0.07
Hematochezia	4(0.83%)	1(7.1%)	3(0.6%)	0.11
Syncope	5(1.04%)	0(0%)	5(1.1%)	0.86
Pulse (mean ±SD)	93.5±10.8	100.4±14	93.3±10.6	0.015
Shock	83(17.3%)	7 (50%)	76(16.3%)	0.001
Hb. (mean ±SD)	9.2±2.4	8.1±2.3	9.2±2.4	0.09
HCT. (mean ±SD)	28.5±7.1	26±6.6	28.5±7.1	0.17
Creatinine (mean±SD)	1.06±0.33	1.24±0.72	1.06±0.31	0.041
Units of blood Transfused. (mean ±SD)	2.1±0.96	3±1..8	2.07±0.86	0.005
Pulse < 100 BP≥ 100	308 (64.2%)	5 (35.7%)	303 (65%)	0.004
Pulse ≥ 100/ BP ≥100	89 (18.5%)	2 (14.3%)	87 (18.7%)	
Pulse ≥100 /BP<100	83 (17.3%)	7 (50%)	76 (16.3%)	

The mean ulcer size was higher (1.57±0.36 cm) in the rebleed group. In the rebleed group ulcer size was 1-2 cm compared to <1cm in the non-rebleed group. The majority (74%) had duodenal ulcers. Most of the patients were in Forest class III (39.8%) as shown in Table 4.

**Table 4:** Comparison of endoscopic findings between rebleed and no rebleed groups.

Characteristic	All patients (n=480)	Rebleed group (n=14)	Non Rebleed group (n=466)	P-value
Ulcer Size (mean ±SD)	1.21±0.30	1.57±0.36	1.2 ± 0.28	0.002
Ulcer Size <1cm	290(60%)	2 (14.3%)	288(61.8%)	0.001
1-2cm	188(39.2%)	10 (71.4%)	178(38.2%)	
>2cm	2(0.41%)	2(14.3%)	0(0%)	
Ulcer Location Gastric	104(21.7%)	6(42.8%)	98(21%)	0.12
Duodenal	354(73.7%)	8(57%)	346(74%)	
Gasrtric+ Doudenal	22(4.6%)	0(0%)	22(100%)	
Forest Class III	191(39.8%)	0(0 %)	191(41%)	0.001
IIc	13(2.7%)	1(7 %)	12(2.6%)	0.84
IIb	38(7.91%)	4(28.6%)	34(7.3%)	0.01
IIa	75(15.62%)	5(35.7%)	70(15%)	0.05
Ib	154(32.08%)	3(21.4%)	151(32.4%)	0.54
Ia	9(1.9%)	1(7.1%)	8(1.7%)	0.63
Ia+Ib	163(34%)	4(28.6%)	161(34.5%)	0.78

Endoscopic procedures were done in 267 out of 480 patients (54.8%); 146/267 (54.7%) received epinephrine whereas 121/267 (45.3%) received epinephrine + mechanical/thermal therapy. As expected, the mean hospital stay was more in the rebleed group as compared to the non-rebleed group (4.86±2.68 days vs. 2.14±0.93 days) as shown in Table 5.

**Table 5:** Treatment and hospital stay comparison between rebleed and no rebleed groups.

Endoscopic procedure	All patients (n=267)	Rebleed group (n=13)	Non Rebleed group (n=254)	P-value
Epinephrine injection therapy	146(54.7%)	9(69.2%)	137(53.9%)	0.21
Epinephrine + Mech./Thermal Therapy	121(45.3%)	4(30.8%)	117 (46 %)	
Mean Hospital Stay in days ± SD	2.22±1.11	4.86±2.68	2.14±0.934	0.000
Index mortality No	478(99.6%)	12 (85.7%)	466(100%)	0.001
Index mortality yes	2(0.4%)	2(14.3%)	0(0 %)	

## Discussion

A total of 480 consecutive patients of acute UGIB of ulcer etiology were studied. 406/480 (85%) of the patients in this study were men, making men the majority of the study group. Our patients were younger compared to contemporary studies and majority were in the third to sixth decade of life with a mean age of 40.9±15.9 years. This is in comparison to other studies by Villanueva et al. [8], Travis et al. [9], Liu et al. [10], Daniela et al. [11], Laursen et al. [12], and Bitar et al. [13] where in the mean age of studied subjects was 64.9±15; 64.9±15.3, 55.3±17.8,66,74, 52±16.8 years, respectively. Most of our patient population 361/480 (75%) was from rural areas but gender was equally distributed between the two groups.

Although only 95 (19.8%) of patients were smokers, equally distributed between two groups (rebleed and non-rebleed group); the mean number of cigarettes smoked per day was significantly more in the rebleed group (16.7±11.5 vs. 8.3±2.9). We could not find any study for comparison. Alcohol consumption and intake of non-steroidal anti-inflammatory drugs, antiplatelets and anticoagulants were not associated with rebleeding risk which is consistent with studies by Wong et al. [14] and Travis et al. [9] whereas Guglielmi et al. [15] found a significant correlation between anticoagulant use and rebleed.

Our patient population being young had less frequency of various co-morbidities with a mean number of 1.7±0.8 co-morbidities. Major co-morbidities were hypertension, coronary artery disease (CAD), DM, and CKD. Various studies have found a significant association between cirrhosis, CKD, malignancy, and congestive cardiac failure (CCF) with risk of rebleeding and mortality [5,9,15,16]; however, we could not find any significant association between co-morbidities and risk of rebleeding.

The main clinical presentation was malena in 341/480 (71%), hematemesis in 54 (11.3%) and hematemesis + malena in 85 (17.7%) patients which corresponds to the study done by Guglielmi et al. [15] who found malena, hematemesis, and both in 60%, 14%, and 18%, respectively.

Although more patients in the rebleeding group in our study had hematemesis (7/14) than in the study by Guglielmi et al. [15], where hematemesis was substantially related with rebleeding, this difference was not statistically significant. At presentation, mean pulse rate was 93±10 and 100.4±14 in non-rebleed and rebleed groups, respectively. Seven patients (50%) in the rebleed group and only 76 (16%) in the non-rebleed group had a shock at presentation, meaning that hemodynamic instability at presentation was a significant predictor for rebleeding. This corresponds to studies by Lauren [12], Wang [14], and Guglielmi et al. [15] but contrasted with a study by Travis et al. [9].

Mean hemoglobin levels of 9.2±2.4 g/dL and 8±2.2 g/dL in the non-rebleed and rebleed groups, respectively; had no significant correlation with rebleeding in our study. Travis et al. [9] and Wang et al. [14] found a significant association with low Hb. at presentation and rebleeding. The patients who rebled received significantly more units of blood transfusions, mean number of 3±1.8 and 2±0.8 in the rebleed and non-rebleed groups, respectively, which correlates with other studies [15,17].

 Regarding endoscopic findings, high mean ulcer size was significantly associated with rebleed; with a mean ulcer size of 1.57± 0.36 cm vs. 1.2±0.28 in the rebleed and non-rebleed group respectively corresponding to other studies [10,15]. Comparable to other studies [8,10,15] frequency of duodenal ulcers was more than gastric ulcers (74% vs. 22%), more gastric ulcers (6/104; 5.7% vs. 8/354; 2.25%) rebled in our study; though not statistically significant.

One of the important predictors of rebleeding is the endoscopic stigmata of ulcers given by the Forest class. We had more number of patients in Forest class III (39.8%) followed by Ib (32%) and IIa (15.6%) which is higher as compared to 29%, 36.4%, and 25% [10,11,15] for class III; 31% and 18.6% for Ib [8,15] and lower as compared to 66% and 32% [8,9] for class IIa, respectively. In other studies, one of the patients in class III rebled in our study whereas only two out of 188 patients rebled in a study by Guglielmi et al. [15].

Seven percent of patients in class IIc comparable to 9.7%; 10.5% in class IIb which is lower than 17%; 6.6% in class IIa, lower than 19.5%; 1.9% in class Ib, lower as compared to 19% and 11% in class Ia, lower than 23% in our study and study by Guglielmi et al. [15], respectively, rebled. In a study by Travis et al. [9]; one out of four (25%) in class IIb and nine out of 68 (13%) in class IIa rebled, which is higher than our study. Overall significant rebleeding was seen in IIa and IIb ulcers.

Endoscopic therapy was given in 267 (55.7%) patients; epinephrine-only therapy in 146(54.7%) and epinephrine+ mechanical/thermal in 121 (45.3%) as per the endoscopist’s discretion. Although more of the patients in epinephrine-only therapy rebled compared to epinephrine + mechanical (nine [69%] vs. four [31%]), it was not statistically significant.

Although we did not randomize endoscopic therapy in our patients; meta-analysis of various studies has shown that monotherapy with epinephrine injection is more effective than medical therapy in patients with high-risk stigmata, but it is inferior to other monotherapies or combination therapy that uses two or more methods [1].

Patients with high-risk stigmata after endoscopic therapy were observed for 72 hours and were continued on high-dose proton pump inhibitor therapy. Fourteen out of 480 patients rebled as per definition; 13 among the high-risk stigmata group and one from the low-risk group (IIC) giving an overall rebleeding rate of only 2.9% compared to 8%, 21%, 7.7%, 10.8%, 13%, and 17%, in other studies [8,9,11,12,15,18], respectively. If calculated for high-risk stigmata only, i.e., 13/267; the rebleeding rate was 4.8%, which is still lower than other studies.

All rebleeders were taken for a second look at endoscopy and endoscopic therapy. Eight patients were managed by second endoscopic therapy whereas six (42.8% among rebleeders, 1.25% of total) patients continued to bleed or rebled after second endoscopic therapy and needed surgery. Two patients died subsequently among bleeders managed by second endoscopic therapy giving overall mortality of 0.4% (2/480) and a mortality of 14.3% (2/14) for rebleeders which is lower than the overall mortality rate of 10%, 5.3%, 3.1% and 8.09% in studies done by Guglielmi et al. [15], Joseph et al. [19], Thomas et al. [20] and Lazăr et al. [11], respectively, and mortality rate of 23.5% among rebleeders by Guglielmi et al. [15].

For multivariate analysis, we put parameters which showed a p-value of less than 0.05 in univariate analysis like the presence of shock, degree of smoking, units of blood transfused, ulcer size and high-risk endoscopic stigmata. However, none of the parameters showed significance possibly because of the overall low rebleeding rate. At this point, we could not develop a new model for predicting rebleed due to our study's smaller number of rebleeders. Applying the Rockall score to our patients showed that the Rockall score of ≥5 has 84% specificity, 57% sensitivity and 98.5% negative predictive value for rebleeding.

## Conclusions

This hospital-based retrospective study found a very low rebleeding rate and mortality compared to the literature. The low rebleeding rate in our study could be explained by the young population, with fewer co-morbidities and a better response to proton pump inhibitors. The significant parameters related to rebleeding included shock at presentation, degree of smoking, units of blood transfused, ulcer size, and high-risk endoscopic stigmata.
